# Subthalamic nucleus and globus pallidus interna influence firing of tonically active neurons in the primate striatum through different mechanisms

**DOI:** 10.1111/ejn.13726

**Published:** 2017-10-20

**Authors:** Asuka Nakajima, Yasushi Shimo, Takanori Uka, Nobutaka Hattori

**Affiliations:** ^1^ Department of Neurology School of Medicine Juntendo University Tokyo Japan; ^2^ Department of Physiology School of Medicine Juntendo University Tokyo Japan; ^3^ Department of Research and Therapeutics for Movement Disorders School of Medicine Juntendo University Tokyo Japan

**Keywords:** basal ganglia, deep brain stimulation, dopamine, Parkinson's disease

## Abstract

Both the subthalamic nucleus (STN) and the globus pallidus pars interna (GPi) are major targets for neuromodulation therapy for movement disorders. An example of such a therapy is deep brain stimulation (DBS). The striatum is the primary target for pharmacological treatment of these disorders. To further our understanding of both the functional relationships among motor nuclei and the mechanisms of therapies for movement disorders, it is important to clarify how changing the neuronal activity of one target, either by medication or by artificial electrical stimulation, affects the other connected nuclei. To investigate this point, we recorded single‐unit activity from tonically active neurons (TANs), which are putative cholinergic interneurons in the striatum, of healthy monkeys (*Macaca fuscata*) during electrical stimulation of the STN or GPi. Both STN stimulation and GPi stimulation reduced the TAN spike rate. Local infusion of a D2 receptor antagonist in the striatum blocked the reduction in spike rate induced by STN stimulation but not that induced by GPi stimulation. Further, STN stimulation induced phasic dopamine release in the striatum as revealed by *in vivo* fast‐scan cyclic voltammetry. These results suggest the presence of multiple, strong functional relationships among the STN, GPi, and striatum that have different pathways and imply distinct therapeutic mechanisms for STN‐ and GPi‐DBS.

## Introduction

The basal ganglia and cerebral cortex are thought to constitute a parallel, closed‐loop system, the so‐called cortico‐basal‐ganglia circuit (Alexander *et al*., 1986). In this model, the subthalamic nucleus (STN) and the globus pallidus pars interna (GPi) are considered the basal ganglia's input and output structures, respectively. The STN receives cortical information both directly and indirectly and receives inputs from brainstem nuclei such as the pedunculopontine nucleus (Kita *et al*., 2014). The STN projection neurons are glutamatergic and send their axons to the GPi, the substantia nigra pars reticulata (SNr), and the pedunculopontine nucleus. The signals transmitted to the GPi/SNr from the STN return to the cerebral cortex via the thalamus.

The GPi receives glutamatergic input from the STN and gamma‐aminobutyric acid (GABA)‐ergic input from the striatum and projects inhibitory GABAergic efferents to the thalamus. In other words, information from the GPi is transmitted to the cerebral cortex indirectly. The STN and GPi show motor‐ and cognition‐related activity in both animals and humans (Dewey & Jankovic, 1989; Reymann *et al*., 2013; Howell *et al*., 2016). Moreover, several studies have reported that changing STN or GPi activity affects cortical activity (Devos *et al*., 2002, 2004; Yang *et al*., 2015), which leads to alterations in movement or behavior (Reymann *et al*., 2013; Piron *et al*., 2016).

However, this summary of the conventional view misses some points that are relevant here. Although anatomical evidence of direct orthodromic connections between the STN or GPi and the striatum (the latter also considered a primary input station of the basal ganglia) is currently weak, it is possible that modulation of both structures (STN or GPi) affects activity in the striatum via thalamic glutamatergic afferents or other pathways. It is important to clarify the effect of modulating STN or GPi activity on striatal neuron activity for several reasons: (i) The striatum is the main pharmacological therapeutic target for movement disorders such as Parkinson's disease (PD) and dystonia. Notably, PD is the second most common neurodegenerative disorder after Alzheimer's disease. The current gold‐standard treatments for PD are levodopa and dopamine agonists, both of which modulate striatal neuron activity through dopaminergic mechanisms. Many previous studies showed that the striatum plays a crucial role in motor control or cognitive behavior and its activity is strongly modulated by dopaminergic inputs from the substantia nigra pars compacta (SNc) of the midbrain (Kawagoe *et al*., 1998). Recent studies have shown that both the STN and the GPi play an important role in regulating dopaminergic neuron activity (Shimo & Wichmann, 2009; Hikosaka *et al*., 2014). (ii) In addition to these pharmacological therapies, high‐frequency–burst electrical stimulation of the STN or GPi is an important therapeutic option for medication‐refractory motor symptoms in PD (STN or GPi) and dystonia (GPi). However, the precise mechanisms of these therapies remain unclear. Exploring the effect of modulating STN or GPi activity on striatal neuron activity should elucidate the functional relationships among these structures and the mechanisms of neuromodulation therapy in human patients.

Two classes of striatal neuron can be identified electrophysiologically: phasically active neurons (PANs) and tonically active neurons (TANs). PANs are GABAergic neurons that project mainly to the globus pallidus external (GPe) and GPi. TANs are putative cholinergic interneurons that send output to influence GABAergic projection neurons within the striatum (Lim *et al*., 2014). Several studies have investigated the effects of STN stimulation on PAN activity using electrophysiology or microarrays (Kita *et al*., 2005; Gubellini *et al*., 2006; Visanji *et al*., 2015), but only one study explored the effect of STN stimulation on TAN activity (Kita *et al*., 2005); this study was performed in only a small population of neurons and used a 100‐Hz stimulation frequency and an electric pulse duration of 300 μs (or a single electric pulse lasting 300 μs) as the stimulation parameters, which are completely different from the stimulation parameters used in clinical settings. Moreover, no study has investigated the relationships between GPi stimulation and TAN activity, and the mechanisms responsible for the effect of STN or GPi stimulation on TAN activity are unknown. Much attention has been paid to the role of TAN activity in PD and dystonia. TAN activity is strongly modulated by dopaminergic inputs from the midbrain, and loss of a balance between acetylcholine and dopamine transmission has been proposed as an important pathophysiological mechanism of PD (Pisani *et al*., 2007; Aosaki *et al*., 2010; Lim *et al*., 2014; Deffains & Bergman, 2015). Moreover, anticholinergic drugs are the first‐line medical treatment for dystonia (Jankovic, 2009). For these reasons, it is important to explore the effect of STN or GPi stimulation on TAN activity to clarify the mechanisms of both pharmacological therapy and electrical stimulation therapy for PD or dystonia.

Therefore, using three healthy monkeys, we explored how changing STN or GPi activity by electrical stimulation, with parameters similar to those therapeutic for human patients, affects the activity of TANs. Electrophysiological and electrochemical recordings provided further information on the functional relationships among these structures.

## Materials and methods

### Experimental animals

We studied three adult, female Japanese monkeys (U, C, and S; *Macaca fuscata,* 5–7 kg). The experimental protocols were approved by the Animal Care and Use Committee of Juntendo University, and all experiments were conducted according to the guidelines set forth in The National Institutes of Health Guide for the Care and Use of Laboratory Animals. The monkeys were kept in individual primate cages with food and water *ad libitum*. Before the start of the experimental phase of the study, the monkeys were trained daily to sit quietly in a monkey chair. During each recording session, monkeys were lightly sedated with ketamine hydrochloride (Daiichi Sankyo Propharma Co., Tokyo, Japan; 0.5–0.75 mg, i.m.) and medetomidine hydrochloride (Nippon Zenyaku Kogyo Co., Fukushima, Japan; 0.01–0.015 mg, i.m.). The head of the animal was fixed in place using head holders, as detailed in the following section, but the rest of the body was not restrained.

### Surgical procedures

Prior to the procedure to affix the head holders and recording chambers to the skull, the monkeys were sedated via the administration of ketamine (4 mg/kg, i.m.) and medetomidine (1 mg/kg, i.m.). General anesthesia was also induced with phenobarbital while monitoring heart rate and the electrocardiogram. Surgical procedures were conducted under aseptic conditions in an operating room. The skull was exposed, and 15–20 titanium screws were inserted and secured with dental acrylic resin. The screws served as anchors for fixation of the head holder and recording chamber. The recording chamber was implanted at stereotactic coordinates (in mm) AP = +9.8 and LM = +6.2 to access the striatum. Based on The Atlas of the Rhesus Monkey Brain (Saleem & Logothetis, 2012), the striatum is at AP = +9 to +29, LM = +7 to +19; the GPi is at AP = +11 to +19, LM = +5 to +13; and the STN is at AP = +10 to +15, LM = +3 to +9. The antibiotic cefazolin (50 mg, i.m.; Astellas Pharma Inc., Tokyo, Japan) was administered pre‐ and post‐surgically.

### Electrophysiological recording

**Figure 1:**
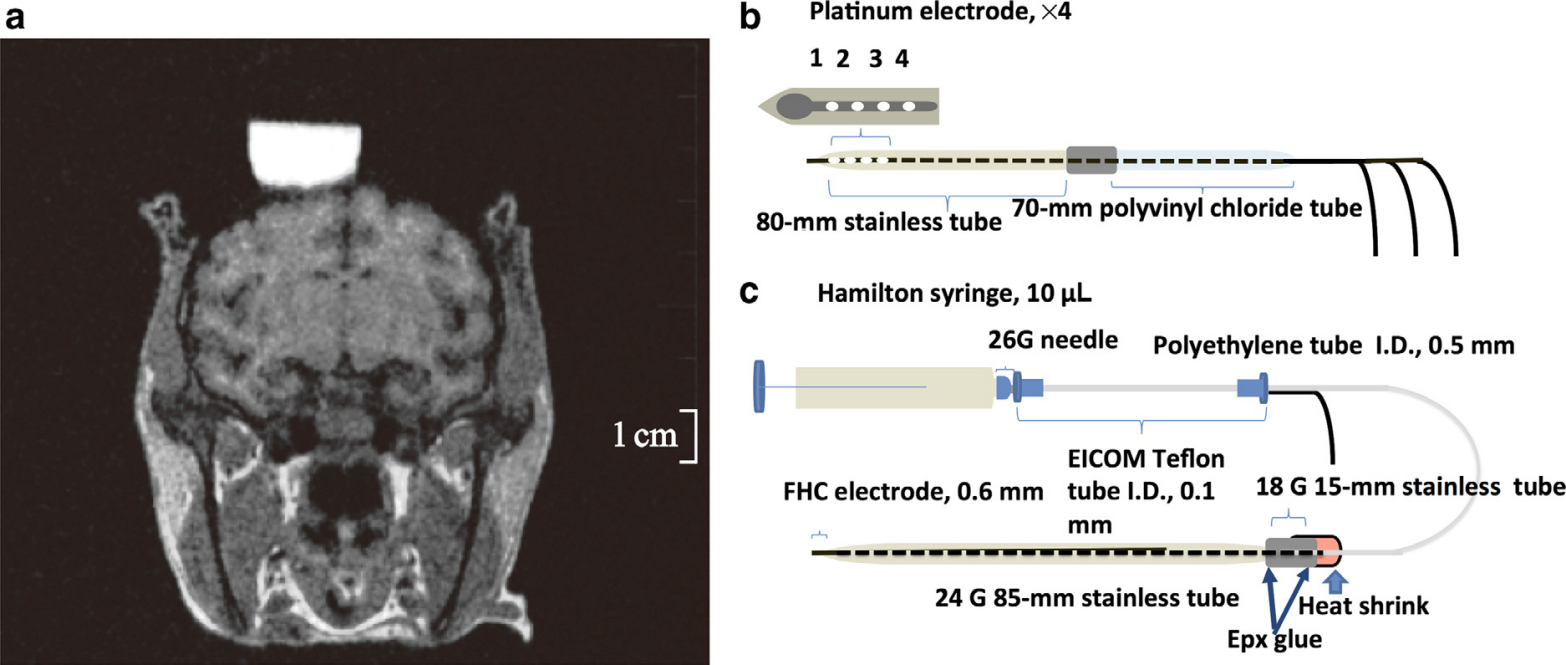
Brain MRI of monkey C (a) and schematic of the experimental setup (b, c). A quadripolar electrode (b) was implanted into the subthalamic nucleus (STN) or globus pallidus interna (GPi). A recording‐injection electrode (c) was inserted into the striatum.

One week after surgery, we obtained magnetic resonance images (0.3 T; AIRIS2, Hitachi Medical Co., Tokyo, Japan) in the plane perpendicular to the recording chamber to estimate the relative positions of the target structures (STN, GPi, and striatum). We recorded single‐unit neuronal activity using tungsten microelectrodes (FHC Inc., Bowdoin, ME, USA) to determine the boundaries of the STN, GPi, and striatum based on these images. During single‐unit recordings, we used a grid system with a 1.0 mm × 1.0 mm spacing (diameter 0.5 mm) to position the recording electrodes or the recording‐injection system in the recording chamber. The microelectrode was inserted through the dura using a guide tube (diameter 24‐gauge). A microdrive (MO903‐A; Narishige, Tokyo, Japan) was used to advance the electrode to the target. The precise position of the STN or GPi was also estimated by their anatomical location as estimated by MRI images (Fig. 1a) and observation of characteristic firing patterns (Rodriguez‐Oroz *et al*., 2001), and by induction of abnormal involuntary movements by local injection of the selective GABA‐A antagonist muscimol (1 μg/μL) to transiently inactivate STN neurons as in previous studies (Kita *et al*., 2006; Shimo & Wichmann, 2009). The location of the stimulation site in the GPi was determined using the same methods as for human patients. The stimulation location was 0.5 mm above the lower GPi border where it is possible to identify noise from the optic tract induced by stimulation of the ipsilateral eye by light flashes. After identifying the location of the location of the STN or GPi, we implanted a custom‐made stimulating electrode (Fig. 1b). The STN and GPi were stimulated by quadripolar electrodes with four uninsulated tips spread 1 mm apart. The length of the STN with this approach was approximately 3 mm. We determined that connecting stimulation contact 1 as the anode and contact 2 as the cathode was optimal for stimulation. The duration of a stimulus current pulse was 60 μs in each phase. The other stimulus parameters were as follows: bipolar stimulation, strength up to 0.4 mA, 130 Hz, delivered for 30 or 10 s. Stimulating parameters such as amplitude, frequency, and anode/cathode contact were determined by observing the response of TAN activity to STN stimulation with several parameters (shown in the Results). The striatum was identified based on its anatomical location, as estimated using MRI images, and position relative to other structures such as the STN and GPi. TANs were identified based on their characteristic spike waveform (broad and often initial positive) and their regular tonic firing pattern (3–10 Hz) that is dissimilar to the very low resting firing frequency of striatal projection neurons, according to previous studies (Aosaki *et al*., 1994a,b; Shimo & Hikosaka, 2001; Morris *et al*., 2004). Recorded signals were amplified (× 8000) and filtered (300–5000 Hz). All waveforms were saved digitally (Multichannel Acquisition; Plexon, Dallas, TX, USA). Individual spikes were distinguished from artifacts using principal component analysis and visualization of the selected waveforms (Off‐line Sort Program, Plexon, Dallas, USA). Briefly, principal component analysis was used to display the recorded waveforms in the 2D Clusters View. Manual sorting methods were used to separate the waveforms including artifacts into individual units. The resulting clusters were inspected, and the units were considered to be separate only if the cluster borders did not overlap. Further, we visually inspected the individual waveforms of the clusters to ensure there were no artifacts. All stimulation and recording positions were tracked using a custom‐made grid system.

### Recording‐injection experiments

To examine the neurotransmitter pathways involved in the regulation of TAN activity, we injected several receptor antagonists locally and subsequently recorded the extracellular activity from TANs during high‐frequency stimulation (HFS) of the STN and GPi. The recording‐injection system consisted of a tungsten microelectrode (FHC Inc.) for unit recording placed within a stainless steel tube (diameter 24‐gauge) for drug delivery (Fig. 1c). This recording‐injection assembly was connected with epoxy resin to polyethylene tubing (inner diameter 0.5 mm), which in turn was attached to Teflon tubing of inner diameter 0.1 mm (EICOM, Tokyo, Japan) leading to a Hamilton microsyringe. The tip of the recording electrode projected 0.25–0.50 mm from the end of the stainless steel tube. During STN and GPi stimulation, we recorded the activity of TANs before, during, and after injection of sulpiride which was adjusted to the final concentration 29 mmol/mL in diluted artificial cerebrospinal fluid (aCSF: pH 7.2–7.4; NaCl, 145; KCL, 2.8; MgCL_2_, 1.2; CaCl_2_, 1.2; and Na_2_HPO_4_, 1; Sigma‐Aldrich, St. Louis, MO, USA; Hadipour‐Niktarash *et al*., 2012), gabazine (1 or 2 mmol/mL in distilled water; Abcam Biochemicals, Cambridge, UK; Galvan *et al*., 2005), or CGP55845 (0.05 or 0.1 mmol/mL in aCSF; Sigma‐Aldrich; Kita *et al*., 2006). The drug solutions were injected through the stainless steel tube by hand pressure applied to the Hamilton microsyringe (0.2 μL per 30 s to a total of 1.2 μL). Previous studies using a similar injection method indicated that the drug‐affected area could be 0.5–1.0 mm in diameter (Shimo & Wichmann, 2009). Moreover, the effects of the drug were evaluated at most 5–10 min after injection (Kita *et al*., 2004). Drugs were not injected until a stable baseline recording of at least 1 min had been acquired, and we continued recording for at least 10 min after the end of the injection. Only one injection was carried out per day.

### Fast‐scan cyclic voltammetry

Carbon fiber microelectrodes cut to an exposed length of 250–300 μm and protruding from stainless steel tubes were connected to a commercial control and recording system (IMEC‐701; Inter Medical Co., Nagoya, Japan) for *in vivo* voltammetry. Immediately before use, the electrode was calibrated *in vitro* for approximately 30 min in dopamine solutions of known concentrations in PBS. Triangular waveforms (−0.4 to 1.5 V relative to an Ag/AgCl reference, 9.3 ms duration, voltage scan rate of 408.6 V/s) were applied to the carbon fiber electrode at 10 kHz for electrochemical recording. Each scan lasted 60 s, and the electrode potential was set to 0 V between scans. This current waveform yielded an oxidant peak for dopamine at 600 mV vs. Ag/AgCl. The stimulation parameters were the same as in the recording‐injection experiments. Stimulus trains lasting for 2 s were delivered starting 30 s after the onset of the 60 s voltammetric recording. Each stimulation pulse train was separated from the previous by a minimum of 5 min. After data collection, background subtraction was performed with commercially available software (EC Analyze, Inter Medical Co.). A color plot of current was used to visualize the data, with the abscissa time and the ordinate applied potential.

### Data analysis

We analyzed TAN activity offline. Spontaneous single‐unit spiking activities were converted to interspike intervals (ISIs) to calculate discharge rates (binned in 500‐ms intervals). To evaluate the responses of individual TANs to STN‐ and GPi‐HFS, ISIs during the 30‐s period before stimulation were compared to ISIs during stimulation.

To evaluate the responses of TANs to drug injection during STN‐HFS and GPi‐HFS, peri‐stimulus time histograms (PSTHs; bin width of 0.5 s) were constructed for stimulus trials. Changes in neuronal activity in response to drug injection before and during stimulation were analyzed using the nonparametric Mann–Whitney *U*‐test.

In addition, using an algorithm based on the Poisson ‘surprise’ method (Legendy & Salcman, 1985), we calculated the occurrence of burst discharges to determine the changes in the TAN firing pattern after drug or electrical stimulation. The algorithm was separately applied to the data that was collected either pre‐ and post‐injection or before and during stimulation. We have previously used this method (Shimo & Wichmann, 2009). Groups of ISIs were compared to the overall distribution of ISIs in the spike train. For the initial classification of a burst, two consecutive ISIs (three spikes) each had to be shorter than half of the cell's mean ISI. In addition, the Poisson ‘surprise’ value for this ‘burst’ [i.e., the negative logarithm of the probability of the occurrence of the sequence of ISIs constituting the burst under the assumption of random (Poisson) firing] had to be ten or greater. In subsequent steps, additional ISIs were added to the beginning or the end of the burst until further extension of the burst did not further increase the resulting burst's surprise value. This burst detection method is frequently used in primate and human recording studies and yields conservative estimates for the occurrence of bursts. The pre‐ and post‐injection burst indices of individual cells were compared using a Mann–Whitney *U*‐test.

For all statistical tests, *P *<* *0.05 was accepted as significant. Discharge rates are expressed as mean ± standard deviation. The recording site for each monkey was reconstructed at the end of experimentation using MRI and the recording results.

## Results

### TAN response to STN or GPi high‐frequency stimulation (HFS)

**Figure 2:**
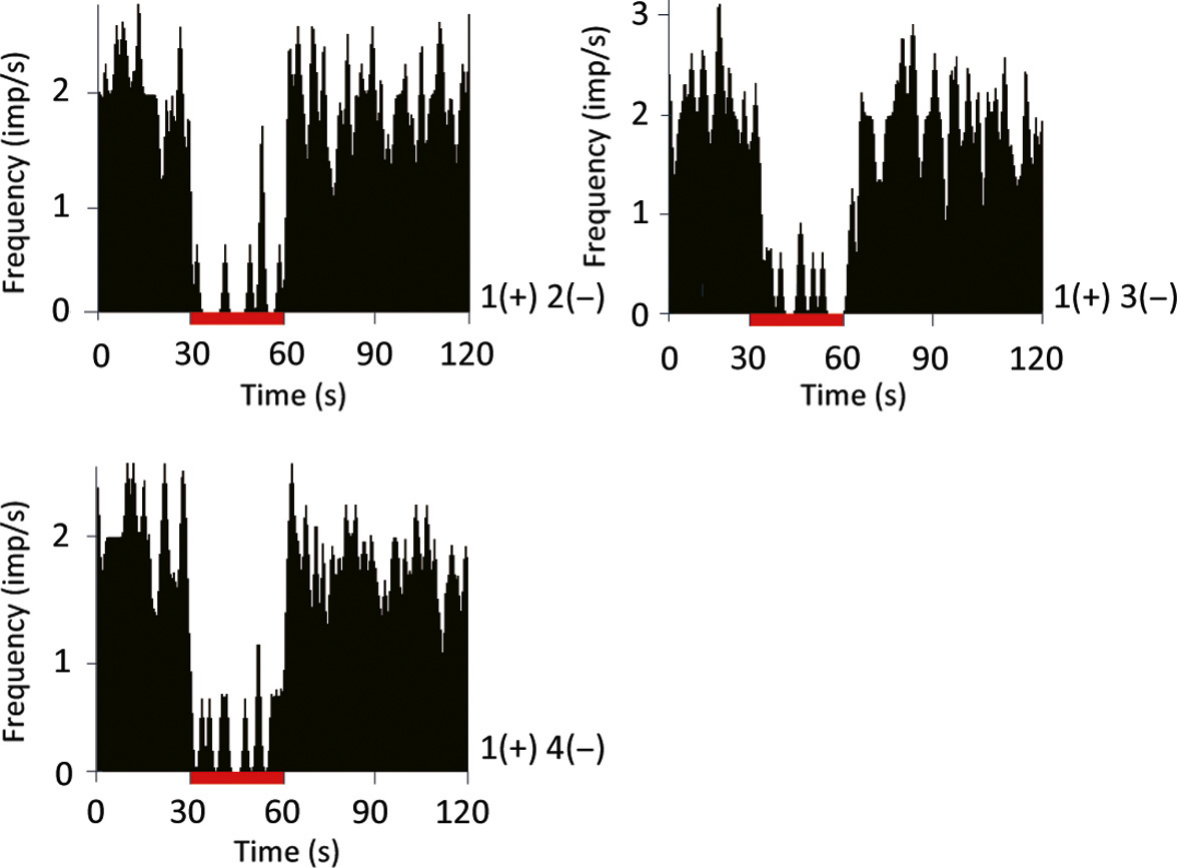
Effect of different subthalamic nucleus (STN) quadripolar stimulating electrode configurations on HFS‐induced suppression of the striatal tonically active neuron (TAN) spike rate. Peri‐stimulus time histogram (PSTH; bin width of 0.5 s) recorded from same TAN neuron. The stimulation lasted 30 s. The tonic firing rate of striatal TANs was suppressed during STN‐HFS using all three quadripolar configurations without significant differences (*P *>* *0.05, Kruskal–Wallis test). Red bars indicate the stimulation period. HFS: high‐frequency stimulation; (+): anodes; (−): cathodes. The location of the stimulation contact is described in Fig. 1. [Colour figure can be viewed at wileyonlinelibrary.com].

**Figure 3:**
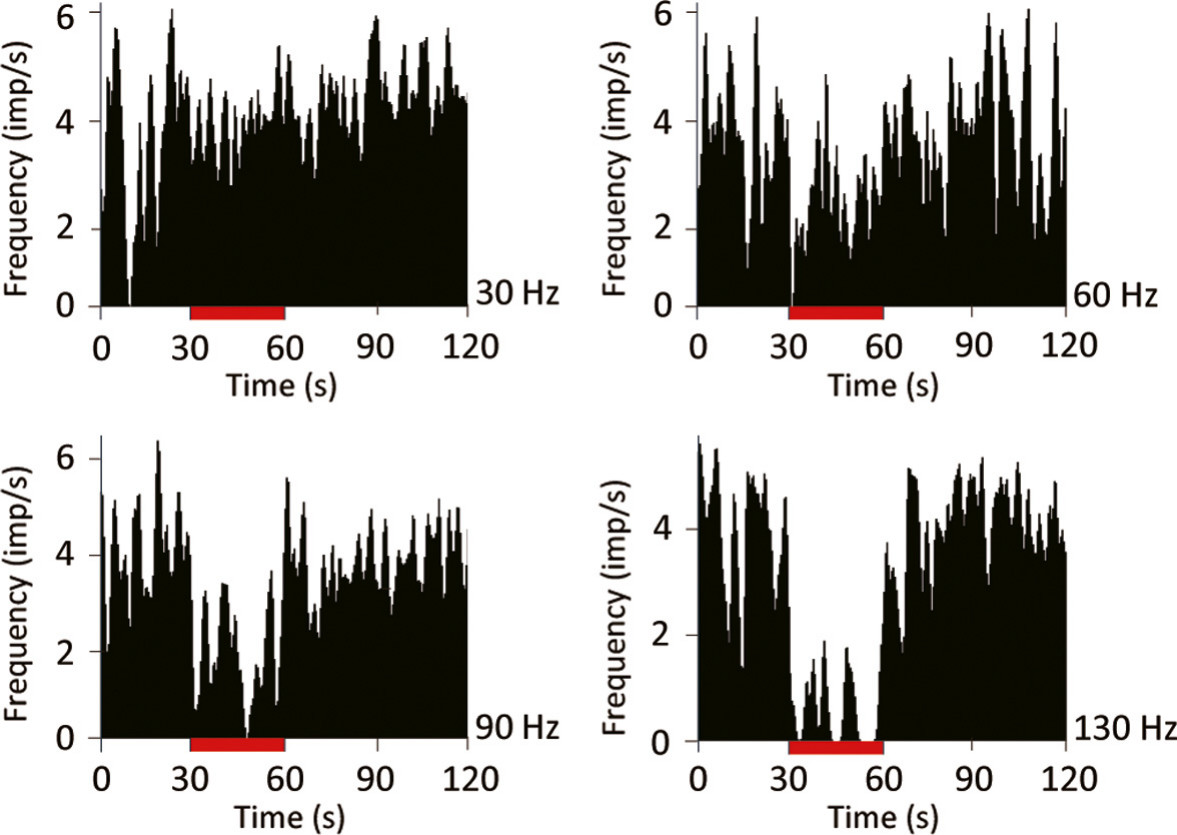
Effect of subthalamic nucleus stimulation frequency on the striatal tonically active neuron (TAN) spike rate. Peri‐stimulus time histogram (PSTH; bin width of 0.5s) recorded from same TAN neuron. The stimulation lasted 30 s. The suppression of TAN firing was measured at multiple stimulation frequencies. The suppression of tonic firing was greatest at 130 Hz (*P *<* *0.05, Kruskal–Wallis test). [Colour figure can be viewed at wileyonlinelibrary.com].

**Figure 4:**
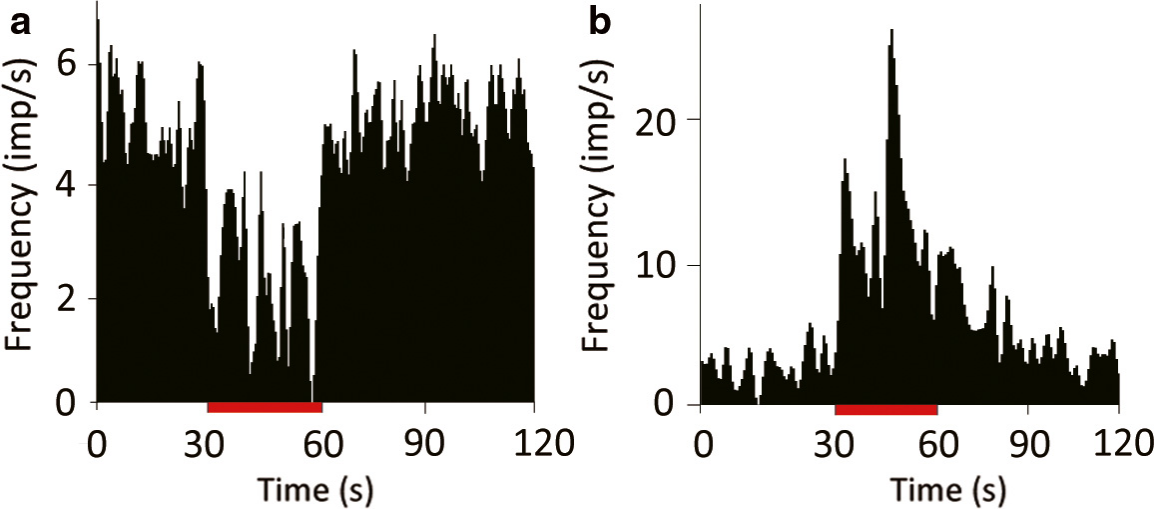
Response variability of TANs to GPi‐HFS. Most TANs showed an 89% decrease in activity to GPi‐HFS (a), but a minority (5%) were excited (b) (*P *<* *0.05, Mann–Whitney *U*‐test). Red bar indicates the stimulation period (30 s). [Colour figure can be viewed at wileyonlinelibrary.com].

To determine the appropriate stimulation parameters, we first tested several configurations of the quadripolar stimulating electrode. We tested all anode–cathode configurations with a fixed current (0.4 mA), frequency (130 Hz), and pulse width (60 μs). All the anode–cathode configurations tested markedly reduced TAN activity with no statistical differences among them [1(+) 2(−); *P *=* *0.017, 1(+) 3(−); *P *<* *0.001, 1(+) 4(−); *P *<* *0.001, Kruskal–Wallis test; Fig. 2]. Next, we determined the appropriate current amplitude for these experiments. We tested 0.1 and 0.4 mA with the same stimulating frequency, pulse width, and configuration (anode, contact 1; cathode, contact 2). The results of this test indicated that the larger amplitude induced a stronger suppression of TAN activity. Moreover, the value of 0.4 mA is consistent with previous reports (Santaniello *et al*., 2012). Higher frequencies of STN stimulation induced a stronger suppression of the TAN spiking rate (30 Hz; *P *=* *0.162, 60 Hz; *P *=* *0.114, 90 Hz; *P *=* *0.05, 130 Hz; *P < *0.001, Kruskal–Wallis test; Fig. 3). From these results, we determined that a stimulus of 0.4 mA, 130 Hz, and 60 μs pulse width from a quadripolar electrode configured with contact 1 as the anode and contact 2 as the cathode reliably modulates TAN spiking activity. Thereafter, we measured the spiking activity of 103 TANs during STN‐HFS (50 from monkey U, 11 from monkey S, 42 from monkey C) and 65 TANs during GPi‐HFS (11 from monkey U, 34 from monkey S, 20 from monkey C). The average resting discharge rate of our TAN population was 4.66 ± 3.22 spikes/s. The majority of TANs showed reduced spiking rates during both STN‐HFS and GPi‐HFS (91% of recorded neurons inhibited during STN‐HFS; 42 from monkey U; 11 from monkey S; 41 from monkey C and 89% during GPi‐HFS; 11 from monkey U; 26 from monkey S; 19 from monkey C; Fig. 4).

### Effect of STN‐HFS on GPi activity

**Figure 5:**
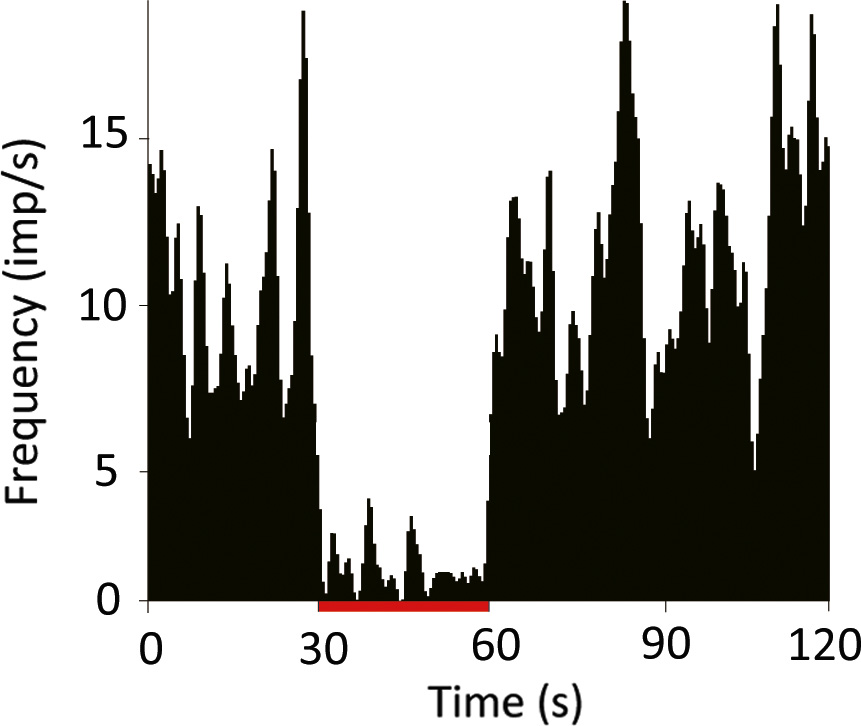
Changes in GPi‐neuron activity in response to STN‐HFS. The spiking activity of most globus pallidus pars interna (GPi) neurons (82%) was reduced by STN‐HFS, while a small minority (12%) showed an increase in excitability (spike rate) (*P *<* *0.05 vs. baseline spike rate, Mann–Whitney *U*‐test). Red bar indicates stimulation period (30 s). [Colour figure can be viewed at wileyonlinelibrary.com].

Next, to clarify the effect of stimulation with our parameters on the projection areas, we tested the effect of stimulation in STN on GPi activity. The average resting discharge rate of 14 GPi neurons (12 from monkey U, 2 from monkey C) was 38.54 ± 15.52 spikes/s. Most GPi neurons were inhibited during STN stimulation (82%), while only a small minority were excited (12%) or showed no effect (6%) (Fig. 5). These results indicate that STN‐HFS with our parameters suppresses neuron activity in the recipients of the direct projections.

### Effect of local D2 receptor blockade on striatal TAN activity during STN‐HFS and GPi‐HFS

**Figure 6:**
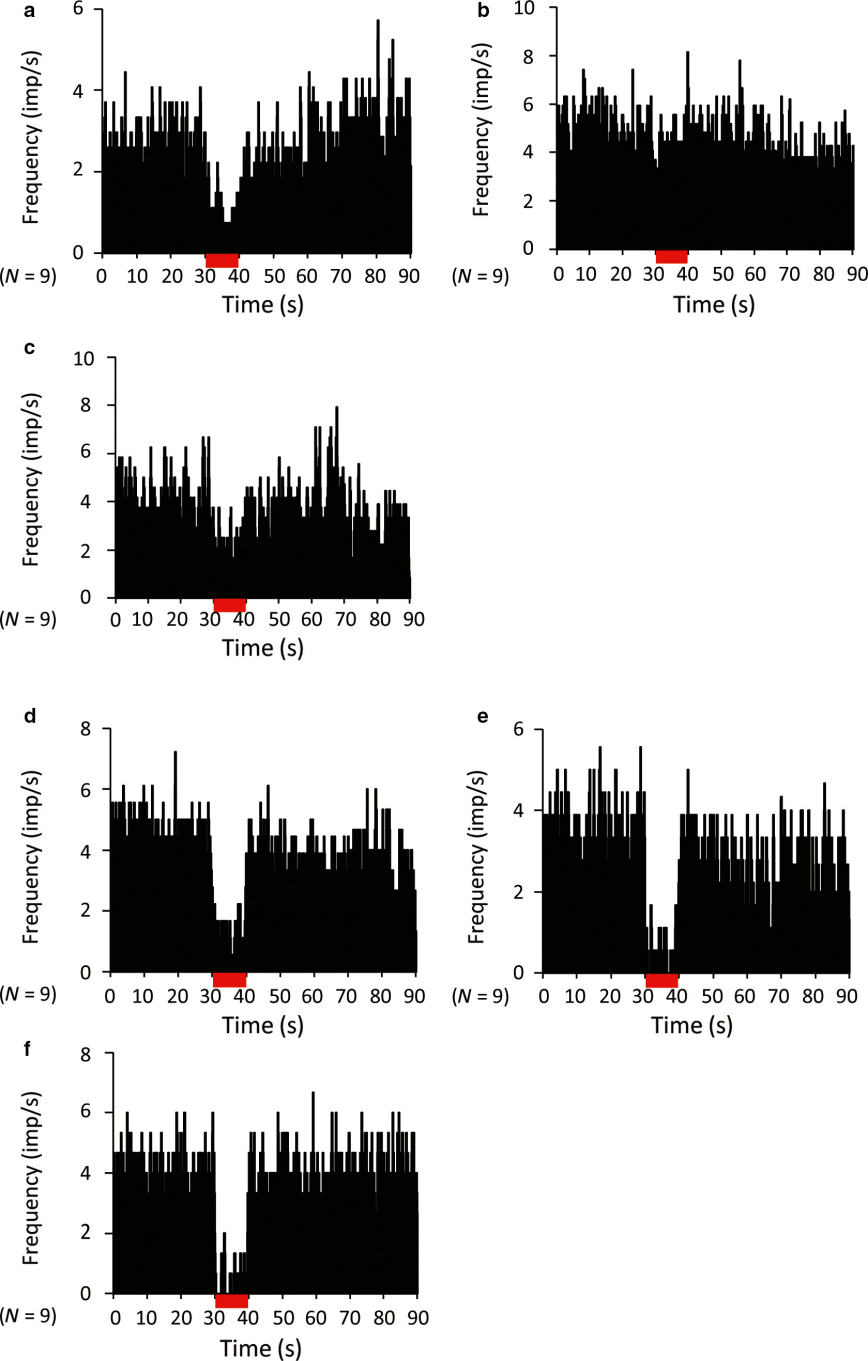
Effect of local injection of the D2 receptor antagonist sulpiride on STN‐ and GPi‐HFS‐induced suppression of TAN firing (population histograms). (a, d) Pre‐injection; (b, e) 2 min after injection; and (c, f) 6 min after injection. The STN‐HFS‐induced suppression of TAN firing was transiently blocked by local injection of sulpiride (a–c). The GPi‐HFS‐induced suppression of TAN firing was not blocked by local injection of sulpiride (d–f). The stimulation lasted 10 s. *N* indicates the number of neurons. [Colour figure can be viewed at wileyonlinelibrary.com].

**Figure 7:**
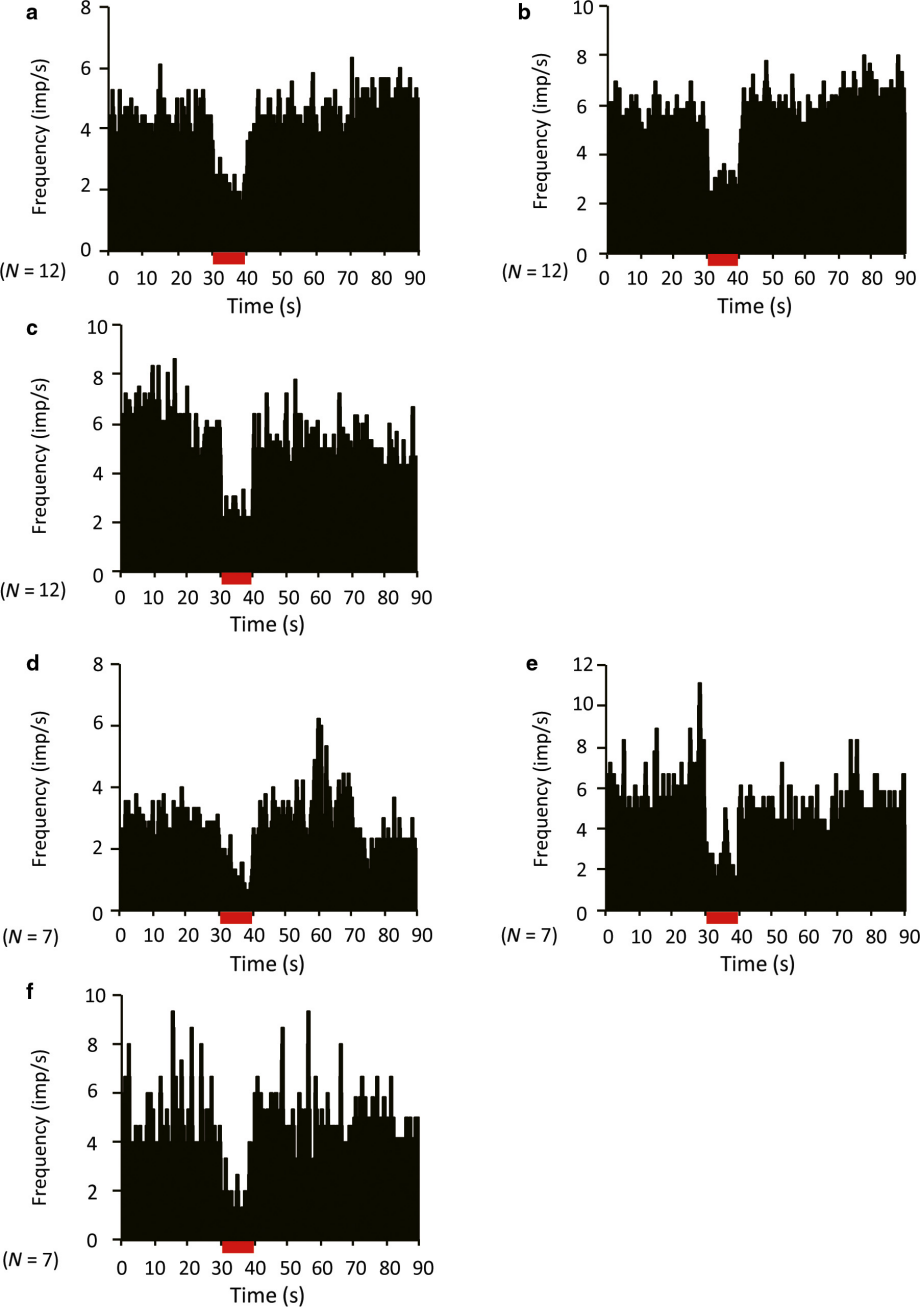
Effect of local injection of the GABA‐A antagonist gabazine or the GABA‐B antagonist CGP55845 on STN‐HFS‐induced suppression of TAN activity (population histograms). (a, d) Pre‐injection; (b, e) 2 min after injection; and (c, f) 6 min after injection. Neither gabazine (a–c) nor CGP55845 (d–f) altered the inhibitory response to STN‐HFS. The stimulation lasted 10 s. *N* indicates the number of neurons. [Colour figure can be viewed at wileyonlinelibrary.com].

Many previous studies have shown that dopamine has a crucial role in modifying TAN activity that is mediated by the D2 receptor. We hypothesized that dopamine contributes to the inhibitory response of TANs during both STN‐HFS and GPi‐HFS. Therefore, we locally injected the D2 antagonist sulpiride and subsequently recorded responses during STN‐HFS and GPi‐HFS. Sulpiride had no effect on both baseline discharge rate (before injection, 6.05 ± 3.89 spikes/s; after, 5.76 ± 3.17 spikes/s; *P *=* *0.941, *N *=* *28, 12 from monkey S, 16 from monkey C) and frequency of bursts [the ratio between the number of discharges in bursts and the total number of action potentials (%). 2.88 ± 2.53 before injection and 5.09 ± 4.14 after injection, *P *=* *0.111]. Before injecting sulpiride, all the recorded TANs reduced their discharge rate during STN‐HFS (6.05 ± 3.89 before stimulation, 1.32 ± 1.07 during stimulation, *P < *0.001). However, at 2 min after injection, sulpiride blocked the STN‐HFS‐induced suppression of discharges (Fig. 7a) in nine of 18 TANs (five of 11 recorded neurons from monkey C and four of seven recorded neurons from monkey S; before stimulation, 4.34 ± 1.55 spikes/s; during stimulation, 4.85 ± 4.63 spikes/s; *P *=* *0.666) without changing the frequency of bursts (4.26 ± 4.89 before injection, 5.52 ± 5.08 after injection *P *=* *0.252; Fig. 6a–c). This effect decayed over several minutes, presumably due to drug diffusion. In contrast, sulpiride had no effect on GPi‐HFS‐induced suppression in any TAN tested (*N *=* *9, 5 from monkey C and 4 from monkey S; before stimulation, 9.20 ± 3.89 spikes/s; during stimulation, 0.93 ± 1.07 spikes/s; *P *<* *0.001) without changing the frequency of bursts (4.57 ± 8.09 before injection, 3.37 ± 4.45 after injection *P *=* *0.856; Fig. 6d–f).

### Effects of GABA antagonists on TAN activity during STN‐HFS and GPi‐HFS

**Figure 8:**
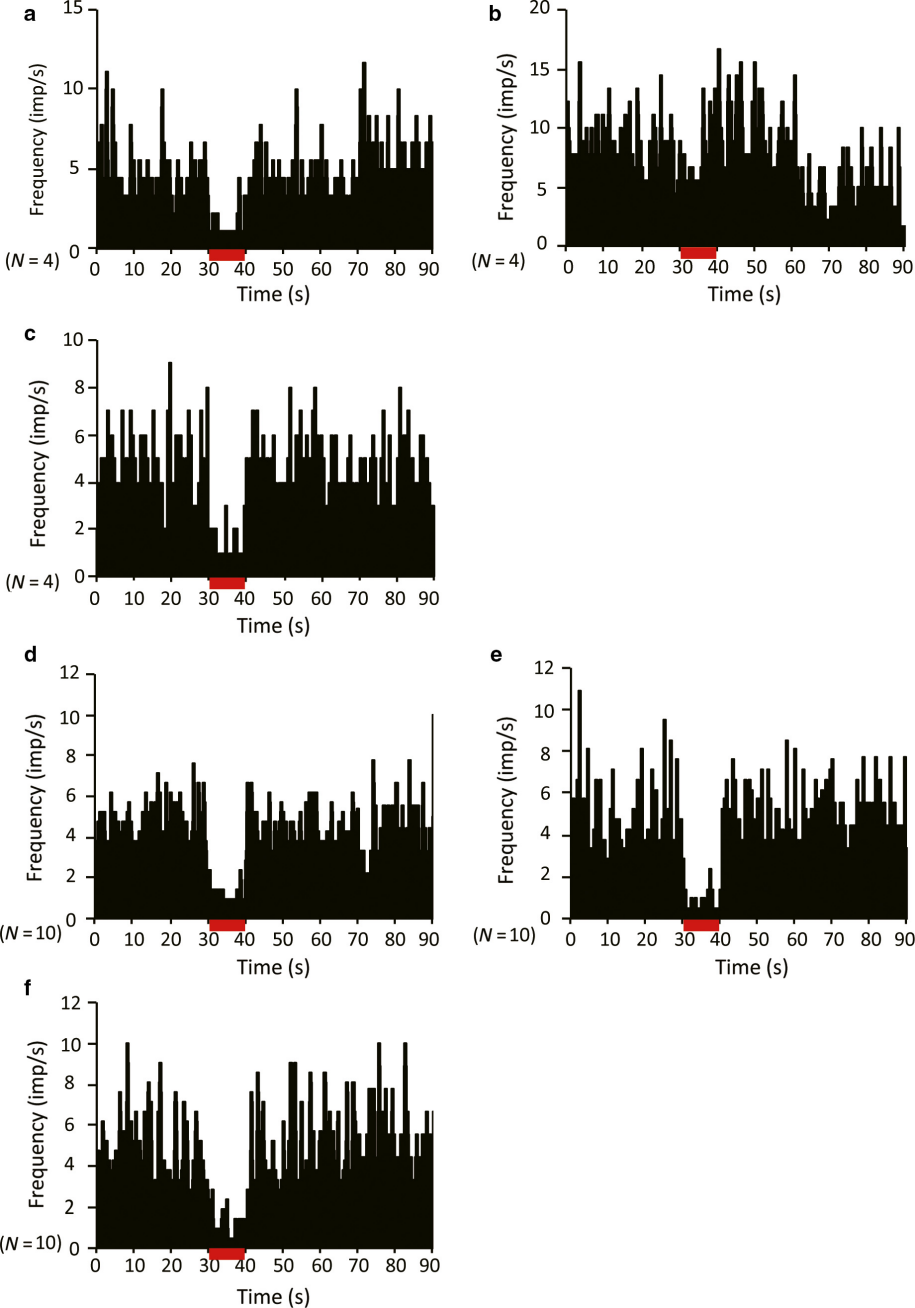
Effect of local injection of CGP55845 or gabazine on GPi‐HFS‐induced suppression of TAN activity. (a) Pre‐injection; (b) 2 min after injection; and (c) 6 min after injection. The GPi‐HFS‐induced suppression of TAN firing was transiently blocked by local injection of CGP55845. This effect was not observed 6 min after injection (a–c). The GPi‐HFS‐induced suppression of TAN firing was not blocked by local injection of gabazine (d–f). The stimulation lasted 10 s. *N* indicates the number of neurons. [Colour figure can be viewed at wileyonlinelibrary.com].

**Figure 9:**
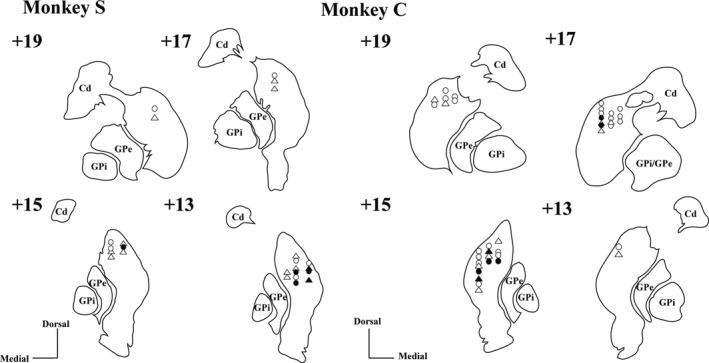
Approximate TAN recording positions in the dorsal striatum of two monkeys reconstructed from coronal MRI images. The location of TANs that were recorded during STN‐HFS (circles) and GPi‐HFS (rectangles) in injection experiments. These locations were identified via reference to an anatomical atlas (Saleem & Logothetis, 2012). Open circles and rectangles indicate the location of TANs that were unaffected by the drugs during STN and GPi‐HFS respectively. The closed circle indicates the location of TANs that responded to sulpiride during STN‐HFS. The closed rectangle indicates the location of TANs that responded to CGP55845 during GPi‐HFS. The number indicates the distance from the interaural plane (mm).

Although the role of GABAergic input in shaping TAN activity is still being debated, recent studies indicate that the inhibition response of TANs may be modulated by GABAergic inputs (Gonzales *et al*., 2013). Therefore, we examined the contribution of GABAergic transmission to TAN responses to STN‐HFS or GPi‐HFS by local injection of the GABA‐A antagonist gabazine or the GABA‐B antagonist CGP55845. Local injection of gabazine did not change either the TAN resting discharge rate (before, 3.75 ± 2.08 spikes/s; after, 4.15 ± 2.15 spikes/s; *P *=* *0.656, *N *=* *22; 14 from monkey C and 8 from monkey S) or the frequency of bursts (3.85 ± 3.96 before injection and 4.11 ± 2.77 after injection, *P *=* *0.714). Similarly, injection of CGP55845 had no effect on either the average TAN discharge rate (before, 6.68 ± 4.12 spikes/s; after, 5.93 ± 3.67 spikes/s; *P *=* *0.687, *N *=* *14; 8 from monkey C and 6 from monkey S) or frequency of bursts (4.82 ± 3.12 before injection and 6.77 ± 4.24 after injection, *P *=* *0.837). Neither gabazine (*N *=* *12; 2 from monkey S and 10 from monkey C; Fig. 7a–c) nor CGP55845 (*N *=* *7; 2 from monkey S and 5 from monkey C; Fig. 7d–f) altered the suppression of TAN activity induced by STN‐HFS. In contrast, CGP55845 did block the inhibitory response to GPi‐HFS in four of seven recorded neurons (two of four from monkey S and two of three from monkey C, *P *=* *0.400; Fig. 8a–c). Injection of gabazine did not show any effect on the inhibitory response of TANs during GPi‐HFS in terms of both discharge rate (before stimulation, 3.54 ± 2.01 spikes/s; during stimulation, 0.53 ± 0.41 spikes/s; *P < *0.001) and frequency of bursts (6.98 ± 7.78 before injection, 3.41 ± 5.95 after injection, *P *=* *0.398; *N *=* *10; 6 from monkey S and 4 from monkey C; Fig. 8d–f). The location of the TANs that were responsive to sulpiride injection during STN‐HFS and CGP55845 injection during GPI‐HFS are shown in Fig. 9. The responsive TANs were mainly located in the dorsolateral region of the putamen and in the region that is posterior to the anterior commissure; these areas correspond to known sensorimotor areas of the putamen (Parent & Hazrati, 1995; Haber, 2016; Marche *et al*., 2017).

### Striatal dopamine release in response to STN‐HFS and GPi‐HFS

**Figure 10:**
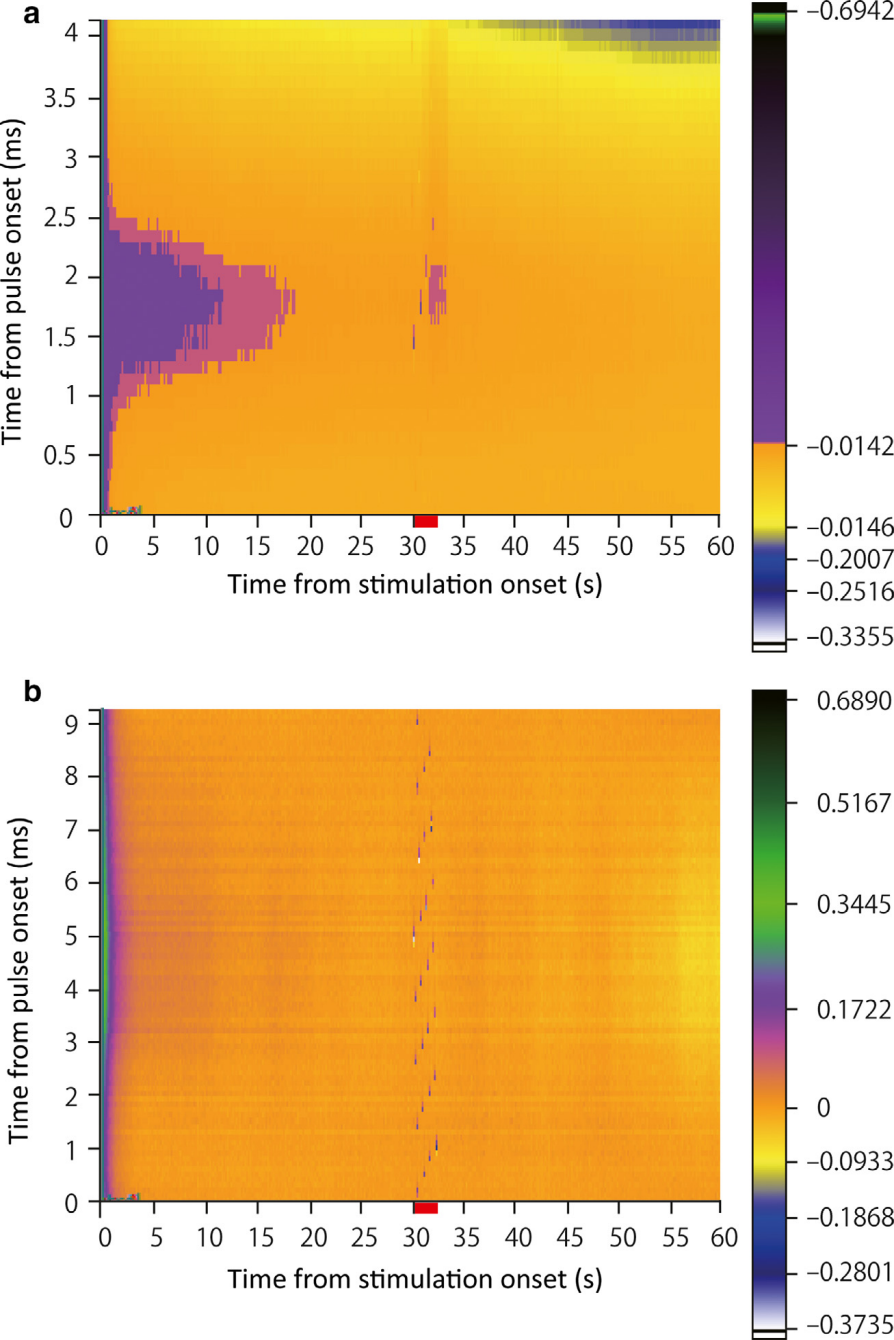
Phasic dopamine release in the striatum is induced by electrical stimulation of the STN but not GPi stimulation. A representative fast‐scan cyclic voltammetry recording, consisting of a background‐subtracted color plot showing a whole −0.4‐ to 1.5‐V triangular waveform. The red bar indicates the stimulating period (2 s). The electrical flow (< 1 nA) corresponds to phasic dopamine release in the striatum after STN stimulation (a). In contrast, an electrical flow indicating phasic dopamine release could not be detected in the striatum during GPi stimulation (b). STN: subthalamic nucleus; GPi globus pallidus pars interna. [Colour figure can be viewed at wileyonlinelibrary.com].

The D2 antagonist sulpiride diminished the inhibitory effect of STN‐HFS on striatal TAN activity but not that of GPi‐HFS, suggesting that STN‐HFS induces dopamine release in the striatum. To confirm this hypothesis, we measured phasic dopamine release during STN‐HFS or GPi‐HFS by *in vivo* voltammetry using a carbon fiber electrode implanted in the striatum. Using the same stimulation parameters as previously described, we found that STN‐HFS induced an electric current in the sub‐nanoampere range, an indicator of phasic dopamine release (in monkey C, *N *=* *4). However, during GPi‐HFS, dopamine release in the striatum could not be detected (in monkey C, *N *=* *5; Fig. 10).

## Discussion

We here demonstrate that electrical stimulation of either the STN or the GPi influences TAN activity. Dopamine is crucial for the TAN response to STN stimulation but not for the response to GPi stimulation, the latter instead being dependent on GABAergic mechanisms. These results indicate strong functional relationships between STN or GPi and striatal neurons, and that dopamine and GABA play important roles in these responses. Moreover, the dopamine dependence of STN‐HFS and dopamine independence of GPi‐HFS may explain the different clinical outcomes of STN‐DBS and GPi‐DBS therapy in PD.

### Functional relationships between the STN and TANs

At this time, we cannot cite clear evidence of direct synaptic connections between the STN and the TANs in the striatum. Therefore, the synaptic pathway mediating TAN inhibition through dopaminergic inputs during STN stimulation is likely to be indirect. Possible candidate pathways are STN‐SNr‐SNc and STN‐GPe‐SNc (GPe, globus pallidus pars externalis). Our previous study identified interactions between the STN and SNc (Shimo & Wichmann, 2009). In rodents, SNc neurons receive inhibitory input from the neighboring SNr (Grace & Bunney, 1979; Celada *et al*., 1999) or from the GPe (Sutton *et al*., 2013). In this study, most GPi neurons showed reduced activity in response to STN stimulation. The mechanism of this remains unclear. Many studies have found that HFS stimulation of the STN mimics the effects of lesioning the STN (Filali *et al*., 2004; Welter *et al*., 2004). Moreover, in non‐human primates, STN‐HFS with similar stimulation parameters induces inhibitory responses in GPi neurons (Kita *et al*., 2005), indicating that STN stimulation may reduce the STN output activity itself, leading to reduction of SNr or GPe activity, which leads to disinhibition of SNc dopaminergic neuron activity by silencing GABAergic axonal collaterals of SNr or GPe to SNc. Another study showed that a distinct group of neurons in the GPi (termed GPh neurons) project to the lateral habenula (Stephenson‐Jones *et al*., 2016). The STN sends axon to the GPh, and the lateral habenula has been shown to directly control the dopaminergic neuronal activity of the SNc (Hong *et al*., 2011). Further, recent anatomical studies identified a direct projection from the STN to the striatum (Koshimizu *et al*., 2013) and the ventral thalamus (Rico *et al*., 2010). This could change TAN activity, thereby triggering dopamine release in the striatum (Threlfell *et al*., 2012). Therefore, several pathways may be responsible for the dopamine‐dependent therapeutic effects of STN‐HFS. Several studies have found evidence supporting our finding of dopamine release during STN‐HFS (Shon *et al*., 2010; He *et al*., 2014; Min *et al*., 2016). In contrast to the influence of dopaminergic D2 receptor signaling, GABAergic transmission did not appear to contribute to the STN‐evoked suppression of TANs. This indicates that STN‐HFS strongly modulates striatal neuron activity by modulating the dopaminergic pathway. In addition to a dopamine pathway, the effects of muscarinic cholinergic autoreceptors on the inhibitory response (Zhang *et al*., 2002) could be an alternative explanation of our results that cannot yet be excluded and requires further examination.

### Functional relationships between the GPi and TANs

The axons of GPi neurons project mainly to the thalamus. Moreover, the thalamus sends glutamatergic efferents to the striatum. Therefore, GPi‐HFS may suppress striatal TAN activity indirectly through thalamic inputs. In this study, we found no evidence of dopamine release during GPi‐HFS; therefore, dopamine is unlikely to contribute to TAN inhibition by GPi‐HFS. A recent study indicates that GPi‐HFS reduces GPi‐neuron activity around the stimulated site, which may lead to increased activity in the thalamus (Chiken & Nambu, 2013), and it is well known that the thalamus sends a massive glutamatergic axonal projection to the striatum (Smith *et al*., 2014). There are two sources of GABA within the striatum. One is the PANs, which send axonal collaterals to striatal neurons, especially to striatal interneurons (Schulz & Reynolds, 2013; Lim *et al*., 2014). The other is GABAergic interneurons, several types of which are present in the striatum (Tepper *et al*., 2010). The precise role of these GABAergic interneurons in the striatum remains unknown, but recent studies have shown that GABAergic interneurons modulate the cholinergic neurons’ activity in the striatum in both normal (Xie *et al*., 2014) and pathological situations (Holley *et al*., 2015) through GABA‐B receptors. From the results of our study, it appears that the disinhibition of thalamic activity by GPi‐HFS may lead to activation of PANs and/or GABAergic interneurons in the striatum, leading to the observed reduction in TAN activity. Another possible mechanism of the effects of GPi‐HFS may be related to the heterogeneity of GPi neurons as discussed above (Stephenson‐Jones *et al*., 2016). Further, TANs receive glutamatergic input from the centromedian–parafascicular complex of the thalamus. This input has an important role in evoking the characteristic response pattern of TANs: a pause followed by an excitatory rebound response (Matsumoto *et al*. 2001). Further experiments are needed to explore the role of glutamatergic input on TAN activity in relation to STN‐ or GPi‐HFS.

### Clinical implications

Because of the therapeutic effectiveness of anticholinergics in PD, it was suggested that disruption of the dopamine–acetylcholine balance in the direction of reduced dopamine and increased acetylcholine is an important factor in PD symptomology (Aosaki *et al*., 2010). Although they account for only 1–2% of striatal neurons, TANs have relatively large cell bodies and send axons extensively to other neurons throughout the striatum. Thus, it is likely that TAN activity has broad effects on striatal function (Girasole & Nelson, 2015). The present study shows that local dopamine D2 signaling is necessary for the STN‐HFS‐induced reduction of TAN activity. This indicates that reduced striatal cholinergic tone in response to dopamine release is a possible therapeutic mechanism of STN‐DBS in human patients. Furthermore, this phenomenon may explain why the dosage of anti‐PD drugs can be reduced in patients after STN‐DBS, but not after GPi‐DBS. Another clinical implication of this study would be an explanation of levodopa‐induced dyskinesia (LID) in human PD patients. Recent studies show a strong relationship between acetylcholine in the striatum and LID. In parkinsonian mice with LID, the basal activity of striatal cholinergic interneurons is elevated (Ding *et al*., 2011). Moreover, ablation of striatal cholinergic interneurons alleviated LID (Won *et al*., 2014). Clinically, both STN‐DBS and GPi‐DBS suppress LID, but through different mechanisms (Follett, 2004). Although the precise mechanisms are unclear, direct modulation effects (both by STN‐DBS and GPi‐DBS) and reduction of dopaminergic drug requirements after STN‐DBS are considered possible therapeutic mechanisms for this phenomenon. From our results, either a direct reduction of cholinergic activity (both by STN‐ and GPi‐HFS) or a release of dopamine in the striatum (by STN‐HFS) can be important in producing this effect.

One important limitation of our study should be mentioned. These results were not obtained in a PD model but rather in healthy monkeys with fully functional DA neurons. 1‐Methyl‐4‐phenyl‐1,2,3,6‐tetrahydropyridine (MPTP) is the gold‐standard toxic agent for establishing the monkey PD model. In patients with PD, however, degeneration of dopamine neurons in the SNc starts in the ‘ventral tier’ and progresses to the ‘dorsal tier’ (Gibb & Lees, 1991). No study has reproduced this degeneration pattern in an MPTP animal model. Although the best time to administer DBS is still in debate, our study suggests that better outcomes will be achieved if it is done before the disease has caused substantial loss of DA neurons. Animal models of PD that replicate the slow progression of neurodegeneration seen in patients will be needed to clarify this point.

In conclusion, we provide evidence that the STN and the GPi each have a functional relationship with the striatum. This study suggests that TANs, which are putative striatal cholinergic interneurons, can be strongly modulated by either STN or GPi activity through different mechanisms, and the results shed light on the reasons for the differential effects of STN‐ and GPi‐DBS in patients with PD.

## Conflict of interests

The authors declare no competing financial interests.

## Author contributions

A.N. and Y.S. made substantial contributions to the study concept and design, acquisition of the data, and analysis and interpretation of the data. A.N., Y.S., T.U., and N.H. participated in drafting the article or critically revising it for important intellectual content. All authors gave final approval of the version to be submitted and any revised version.

## Data accessibility

All raw data are stored in the Department of Neurology, Juntendo University School of Medicine, and are available upon request to the corresponding authors.

**Abbreviations**
DBS: deep brain stimulation
GABA: gamma‐aminobutyric acid
GPi: globus pallidus interna
HFS: high‐frequency electrical stimulation
ISI: interspike interval
LID: levodopa‐induced dyskinesia
MPTP: 1‐methyl‐4‐phenyl‐1,2,3,6‐tetrahydropyridine
PAN: phasically active neuron
PD: Parkinson's disease
SNc: substantia nigra pars compacta
SNr: substantia nigra pars reticulata
STN: subthalamic nucleus
TAN: tonically active neuron
