# Supplementary material for: Subthalamic nucleus and globus pallidus interna influence firing of tonically active neurons in the primate striatum through different mechanisms
Source: Eur J Neurosci. 2017 Oct 20;46(11):2662–73. doi: 10.1111/ejn.13726 (PMC5765455; doi:10.1111/ejn.13726)
Supplement: Supplementary file 1 [file EJN-46-2662-s001.pdf]

EJN European Journal of Neuroscience

FENS Federation of European Neuroscience Societies

Subthalamic Nucleus and Globus Pallidus Interna Influence Firing of Tonically Active Neurons in the Primate Striatum through Different Mechanisms

Asuka Nakajima, Yasushi Shimo, Takanori Uka & Nobutaka Hattori

Review timeline:

Submission date:	24 March 2017
Editorial Decision:	28 April 2017
Revision received:	21 July 2017
Editorial Decision:	21 August 2017
Revision received:	19 September 2017
Accepted:	21 September 2017

Editor: Yoland Smith

1st Editorial Decision 28 April 2017

Dear Dr. Shimo,

Your manuscript was reviewed by external reviewers as well as by the Section Editor, Dr. Yoland Smith, and ourselves. The reviews collectively indicate that your experiments generated new and important information. However, there are several issues that need to be clarified/resolved before we can consider your manuscript further for publication in EJN.

Both reviewers agree that the question under study in your manuscript is relevant. However, they raise significant concerns about the level of rigor in the data collection and analysis and the lack of key methodological details needed to understand the study design and interpret your data. They also raise a concern about the lack of convincing rationale to focus on cholinergic interneurons instead of striatal projection neurons. They both raise important suggestions to improve the quality of the figures and readability of the manuscript. Please, make sure to carefully address each of their suggestions in the revised manuscript.

We also note the following points:
- Please ensure that the reporting of statistical data adheres to EJN guidelines, notably please report the precise values of P.
- Data statement needed
- Larger, better resolution figures are needed

If you are able to respond fully to the points raised, we would be pleased to receive a revision of your paper within 12 weeks.

Thank you for submitting your work to EJN.

Kind regards,

Paul Bolam & John Foxe
co-Editors in Chief, EJN

Reviews:

Reviewer: 1 (Paul Apicella, Aix Marseille Université, France)

Comments to the Author
This is a manuscript that deals with an interesting issue: what are the neural bases of behavioral effects of high frequency electrical stimulation (HFS) of the STN and GPi in nonhuman primates. To examine this issue, the authors have recorded neuronal activity from the striatum of awake macaque monkeys, focusing on the so-called TANs, thought to be cholinergic interneurons (ChIs). A major finding of this work is that the

"spontaneous" firing rate of striatal ChIs can be modulated by either STN or GPi HFS, but these effects appear to rely on different mechanisms. Notably, depression of ChI firing in response to STN stimulation was dependent on dopamine (DA) input to the striatum, whereas ChI depression elicited by GPi stimulation was not linked to striatal DA transmission. As demonstrated in this study, local injection of a D2 receptor antagonist in the striatum blocked the reduction in TAN firing elicited by STN HFS but not that induced by GPi stimulation. This leads the authors to the interesting suggestion that "reduced striatal cholinergic tone in response to DA release is a possible therapeutic mechanism of STN-DBS in human patients". In this regard, the last part of the Discussion ("Clinical implications") offers promising prospects for further studies of the relationship between STN HFS and striatal DA transmission. Although the overall question this study asks is an interesting one, the analysis does not go far enough and there are a number of problems with this manuscript that must be addressed. In particular, there is a lack of care in providing accurate information on the data. To say the least, the present version is not a carefully written report and this undermines the potential interest of the study

Major
1. Although the issue examined in this study is interesting, the results are not clearly presented and the present version is not satisfactory, with a flagrant lack of detailed information. In the Introduction, it is unclear why the authors put such a strong emphasis on the ChIs, instead of striatal output projection neurons (MSNs). If they think that it would be desirable to clarify the effect of STN- or GPi-HFS on striatal neuron activity, one wonder why they don't examine MSN activity.

2. There is a lack of rigour in the description of the results in several places in the text. It would be valuable to make changes in the manuscript to improve its clarity and readability, particularly in the Results section which is difficult to follow. It needs to be tightened and made clearer to allow the reader to focus on the important findings with all relevant details. The present version is somewhat over-illustrated with repetitive Figures which do not appear to be prepared as thoroughly as possible :
- Fig.2: This Fig is not informative. Where are recording sites located ? It's very hard to recognize the putamen on these small drawings. The shape of the putamen appears different from one animal to the other (e.g., compare +19 in monkey S to +19 in monkeys U and C). There is a lack of information about the levels indicated (interaural plans ?)
- Figs. 3, 4: Is it the activity of 3 distinct TANs or the same TAN ? Move info about binwidth in the caption. Be more explicit about the information provided (1(+)2(-), etc...)
- Fig. 5 and 9: poor quality, scales not visible
- Fig. 6: Why is the red bar inserted in the PETH ?
- Figs. 7, 8: What are the Ns ? Why do you mention abbreviations which are not used in the Fig ? (GPi, HFS, STN, TAN)

3. I am missing details of the recorded TAN population, i.e., number of neurons recorded per monkey. We would need to know if the measures for TAN responses to STN-HFS are reliable between the 3 monkeys. It seems to me that the sample size of tested neurons is quite small in some instances (e.g., pharmacological tests). Also, there is a very limited amount of analysis based on simple comparisons of spontaneous firing rates 30 s before and 30 s during HFS. What about possible changes in firing patterns ?

4. The authors did not refer to the locations of recorded neurons shown in Fig. 2. They should describe the distribution of recorded neurons in the different stimulation protocols (STN-HFS and GPI-HFS). Are they located in the sensorimotor area of the putamen? These data should be provided.

Minor
Abstract
missing word « To further (?) our understanding of both the functional relationship... »

Methods
No sufficient care is taken to describe the behavioral condition in which HFS was applied. Is the monkey supposed to behave in a certain way ? Waiting for a stimulus or reward ? body posture ? arm/hand position ?
p.9: the reference of the atlas of Saleem & Logothetis is not necessary in the text (also in the caption of Fig.2)
p.11: more information is needed about the identification of TANs to be included in the Results section.
p.13: Fast-scan cyclic voltammetry needs to be checked by an expert
p.14: typo: « Spontaneous »

Results
p.15: « Response of TAN activity to STN or GPi high-frequency stimulation » TAN response to... or change in TAN activity in response to...
p.19: information about suppliers should be put in materials & methods
Fig.1: Is it possible to improve the quality of the brain scan ?

Discussion
p.20 "Dopamine is crucial for the response to STN stimulation but not for the response to GPi stimulation..." Do you mean the TAN response to STN stimulation ?
p.21 "... the relative efficacies of these two DBS targets will prove to be a strong function of disease progression (i.e., the extent of dopaminergic degeneration)" this sentence seems incorrect.
p.23: « PANs send axonal collaterals to striatal neurons, especially to TANs » should be: to striatal interneurons
p.23 "The precise role of this neuron (which one ?) in the striatum remains unknown, but recent studies have shown that GABAergic interneurons"...

Abbreviations: typo: MPTP; 1-methil-4-phenyl-1,2,3,6-tetrahydropyridine

References must be checked. Some of them are not properly formatted.

Reviewer: 2 (Natalie Doig, University of Oxford, UK)

Comments to the Author
This study examines the relationship between the subthalamic nucleus, the globus pallidus interna and striatal putative cholinergic interneurons using high frequency stimulation in non-human primates. As the STN and GPi are the major targets for DBS in PD patients the authors use similar stimulation parameters and recorded from TANs in the striatum. The authors also use pharmacological tools and show that the decrease in TAN firing is dependent on the D2 receptor following STN HFS but not GPi HFS; whereas GPi HFS was shown to be a GABAergic mechanism via GABA-B. Cholinergic interneurons (presumably TANs recorded here) have known to be involved in the pathology of PD. This study concisely describes a link between DBS and changes in TAN firing and highlights some potential mechanisms.

Specific Comments
1. It would be beneficial to include a figure or a panel in figure 3 to show an example of the firing of an individual TAN in response to the stimulation.
2. It would also be interesting to see the response of PANs as a comparison?
3. Were all combinations of pharmacological experiments carried out? In particular, was the GPi-HFS examined in the presence in of a GABA-a antagonist?
4. General comment on figures – there is some irrelevant information on the figures. For example, bin size could go in the legend; and the text on the axes could be larger.
5. The discussion needs to be expanded to consider other possible mechanisms. For example:
o A study has shown that STN neurons can directly innervate neurons in the CPu (Koshimizu et al., 2012) which could explain the proportion of TANs that increase their firing rate in response to HFS. And this could also explain increase in levels of dopamine as in Threlfell et al., 2012.
o As well as antidromic stimulation of the thalamus via the GPi HFS, STN HFS could also cause antidromic thalamic stimulation which could contribute to the mechanisms described here.
o GPi HFS could lead to the stimulation of multiple neuron types as described in Stephenson-Jones et al., 2016 which could cause a variety of effects.

Authors' Response 21 July 2017

Response to the reviewer
We thank the reviewers for their careful reading our manuscript and their valuable comments to improve the quality of our manuscript. We have revised the manuscript based on the reviewer comments.

Major concerns from Reviewer 1:

Although the issue examined in this study is interesting, the results are not clearly presented and the present version is not satisfactory, with a flagrant lack of detailed information. In the Introduction, it is unclear why the authors put such a strong emphasis on the ChIs, instead of striatal output projection neurons (MSNs). If they think that it would be desirable to clarify the effect of STN- or GPi-HFS on striatal neuron activity, one wonder why they don't examine MSN activity.
Response: We thank the reviewer for this comment. We added several sentences to the Introduction to clarify why we examined the response of TANs. Our changes to the manuscript are expressed in underlined text. Several studies have investigated the relationships between MSN activity and STN or GPi modulation and only one study investigated TANs in relationship to the modulation of STN activity with different from

clinical stimulation parameters with small sample size. Therefore, exploring TAN activity changes during STN- and GPi-HFS with almost same to clinical stimulation settings will reveal new information about the therapeutic mechanisms of DBS.

There is a lack of rigour in the description of the results in several places in the text. It would be valuable to make changes in the manuscript to improve its clarity and readability, particularly in the Results section which is difficult to follow. It needs to be tightened and made clearer to allow the reader to focus on the important findings with all relevant details. The present version is somewhat over-illustrated with repetitive Figures which do not appear to be prepared as thoroughly as possible:
Response: We rewrote the results section to address the reviewers concern. We hope that this revised version is more readable and clearer.

Fig.2: This Fig is not informative. Where are recording sites located ? It's very hard to recognize the putamen on these small drawings. The shape of the putamen appears different from one animal to the other (e.g., compare +19 in monkey S to +19 in monkeys U and C). There is a lack of information about the levels indicated (interaural plans ?)
Response: The shape of the putamen from each monkey was reconstructed from an MRI scan of each monkey. Each number (e.g., +13 or +15) represents the distance from the interaural line. Considering the reviewer's comment below about Figure 2, we deleted this figure and include a new figure that shows the location of the neurons that were recorded during the drug injection study (i.e., Figure 9). In this new figure, we also show the location of the neurons that were recorded to show the effects of drugs in response to STN- or GPi-HFS.

Figs. 3, 4: Is it the activity of 3 distinct TANs or the same TAN ? Move info about binwidth in the caption. Be more explicit about the information provided (1(+)2(-), etc...)
Response: Each figure represents the response of one neuron. To clarify this point, we changed the Figure 3 legend to "Effect of different subthalamic nucleus (STN) quadripolar stimulating electrode configurations on HFS-induced suppression of a striatal tonically active neuron (TAN) spike rate." and the Figure 4 legend to "Effect of STN stimulation frequency on a striatal tonically active neuron (TAN) spike rate." The bin width is 500 ms and we added this information to the caption.
The annotations 1, 2, 3, and 4 indicate the location of the stimulating contact (i.e., the unsealed area of the tip of the stimulating electrode). We added more information about the stimulating electrode in Figure 1 to clarify this point. The "+" indicates cathode and the "-" indicates the anode.

Fig. 5 and 9: poor quality, scales not visible
Response: We included higher qualities images for both figures.

Fig. 6: Why is the red bar inserted in the PETH?
Response: We moved the red bar under the abscissa.

Figs. 7, 8: What are the Ns ? Why do you mention abbreviations which are not used in the Fig ? (GPi, HFS, STN, TAN)
Response: Both figures are population histograms of the responsive neurons. The "N" indicates the number of neurons that are included in these histograms. We added the number of neurons that was recorded the experiment. We deleted all abbreviations which are not used in the Figure.

I am missing details of the recorded TAN population, i.e., number of neurons recorded per monkey. We would need to know if the measures for TAN responses to STN-HFS are reliable between the 3 monkeys. It seems to me that the sample size of tested neurons is quite small in some instances (e.g., pharmacological tests). Also, there is a very limited amount of analysis based on simple comparisons of spontaneous firing rates 30 s before and 30 s during HFS. What about possible changes in firing patterns?
Response: Thank you for your helpful suggestion. We performed additional experiments to increase the neuron sample size and state the number of recorded neurons for each monkey in the Results section. Unfortunately, one monkey died; therefore, we only added data from two monkeys for the injection study. We also analyzed the frequency of TAN bursts before and after injection and before and during STN or GPi stimulation to observe firing pattern changes. However, no statistically significant change was observed after drug injection or stimulation. We added these data to the Results section and include information about our burst detection algorithm in the Methods.

The authors did not refer to the locations of recorded neurons shown in Fig. 2. They should describe the distribution of recorded neurons in the different stimulation protocols (STN-HFS and GPI-HFS). Are they located in the sensorimotor area of the putamen? These data should be provided.
Response: We thank the reviewer for this comment. We added information about the location of the TANs that showed a response to drugs during STN- or GPi-HFS in Figure 2. In addition, to address this point, we renumbered "Figure 2" as "Figure 9", as this change made it easier to follow our results. Due to this change, previous figures were also renumbered. As a result of these changes, we clearly show that the responsive TANs tended to be located within a lateral region of the putamen.

Minor concerns from Reviewer 1:
Abstract
missing word « Further to (?) our understanding of both the functional relationship... »
Response: We have changed this sentence as "To further our understanding…."

Methods
No sufficient care is taken to describe the behavioral condition in which HFS was applied. Is the monkey supposed to behave in a certain way? Waiting for a stimulus or reward? body posture ? arm / hand position?
Response: We added information about the condition of the monkey during HFS as "The head of the animal was fixed, but the body could move freely." In the "Experimental animals" section.

p.9: the reference of the atlas of Saleem & Logothetis is not necessary in the text (also in the caption of Fig.2)
Response: We deleted this information from both the text and figure.

p.11: more information is needed about the identification of TANs to be included in the Results section.
Response: We added references about previous studies on TANs to the Results section. We used the same criteria as these earlier studies to identify TANs.
p.13: Fast-scan cyclic voltammetry needs to be checked by an expert
Response: We performed our voltammetry experiments with the assistance of Genko Oyama, a voltammetry expert. Genko Oyama is a co-author and first author of two respective articles on voltammetry. We added his name to the Acknowledgements section of our revised manuscript.

p.14: typo: « Spontaneous »
Response: We corrected this typo.

Results
p.15: « Response of TAN activity to STN or GPi high-frequency stimulation » TAN response to... or change in TAN activity in response to...
Response: We changed to "TAN response to STN or GPi high-frequency stimulation (HFS) "

p.19: information about suppliers should be put in materials & methods
Response: We added this information to the Materials and methods.

Fig.1: Is it possible to improve the quality of the brain scan?
Response: We changed this MRI image to a better quality one.

Discussion
p.20 "Dopamine is crucial for the response to STN stimulation but not for the response to GPi stimulation..." Do you mean the TAN response to STN stimulation?
Response: Yes, we do. To clarify this, we add "TAN response" in this sentence.

p.21 "... the relative efficacies of these two DBS targets will prove to be a strong function of disease progression (i.e., the extent of dopaminergic degeneration)" this sentence seems incorrect.

EJN European Journal of Neuroscience FENS Federation of European Neuroscience Societies

Response: Thank you for pointing this out. Yes, this sentence is incorrect. We changed the sentence to "Moreover, the dopamine -dependence of STN-HFS and -independence of GPi-HFS may explain the different clinical outcomes of STN-DBS and GPi-DBS therapy in PD."

p.23: « PANs send axonal collaterals to striatal neurons, especially to TANs » should be: to striatal interneurons
Response: We changed this sentence.

p.23 "The precise role of this neuron (which one?) in the striatum remains unknown, but recent studies have shown that GABAergic interneurons"...
Response: We changed "this neuron" to "GABAergic interneurons."

Abbreviations: typo: MPTP; 1-methil-4-phenyl-1,2,3,6-tetrahydropyridine
Response: We corrected this typo.

References must be checked. Some of them are not properly formatted.
Response: We reformatted the references with Endnote X10.

Comments of Reviewer 2

It would be beneficial to include a figure or a panel in figure 3 to show an example of the firing of an individual TAN in response to the stimulation.
Response: This figure shows an example of TAN activity during STN stimulation.

It would also be interesting to see the response of PANs as a comparison?
Response: We thank the reviewer for the above comment. We also think it is interesting that we observed an effect of STN or GPi stimulation on PANs; however, several studies have already performed this experiment (albeit different techniques and species were used). Therefore, the aim of our present study was to be the first to explore the effect of STN/GPi modulation on distinct types of striatal neurons. We added a sentence to the Introduction about why we explore TAN activity. Technically, PANs are difficult to identify using our experimental conditions because PANs rarely fire in sedated monkeys. PANs fire phasically during behavior as shown previously (Kimura et al 1986, Kawagoe et al 1998). However, our experimental design did not involve a behavioral task or cortical stimulation like that of Kawagoe et al or Kita et al.

Were all combinations of pharmacological experiments carried out? In particular, was the GPi-HFS examined in the presence in of a GABA-a antagonist?
Response: Thank you for your important question. We performed additional experiments using different combinations of pharmacological treatments with each monkey (see the Results). The TAN discharge response to GPI-HFS was not different before and after injection of a GABA-a antagonist in monkeys. Only STN-HFS plus sulupiride and GPI-HFS plus a GABA-b antagonist affected the TAN response in monkeys.

General comment on figures – there is some irrelevant information on the figures. For example, bin size could go in the legend; and the text on the axes could be larger.
Response: We corrected this figure.

The discussion needs to be expanded to consider other possible mechanisms. For example:
1) A study has shown that STN neurons can directly innervate neurons in the CPu (Koshimizu et al., 2012) which could explain the proportion of TANs that increase their firing rate in response to HFS. And this could also explain increase in levels of dopamine as in Threlfell et al., 2012.

2)As well as antidromic stimulation of the thalamus via the GPi HFS, STN HFS could also cause antidromic thalamic stimulation which could contribute to the mechanisms described here.

3) GPi HFS could lead to the stimulation of multiple neuron types as described in Stephenson-Jones et al., 2016 which could cause a variety of effects.
Response: We thank the reviewer for these important suggestions to improve our discussion. We discuss the relevance of a direct projection from the STN to the CPu and the release of dopamine that cholinergic interneurons trigger. In addition, we discuss the GPh-Habenula-SNc dopamine pathway. However, the second point here from reviewer 2 is not clear to us. Both GPi- and STN-HFS may cause "orthodromic" stimulation (disinhibition) of the thalamus, but unlikely to cause antidromic stimulation of the thalamus. It has been

showed that there is direct projection from the STN to the ventral thalamus (Rico et al 2010). This may cause orthodromic modulation of the thalamic neurons by STN-HFS. We now add this point in discussion.

We believe our changes to the manuscript in response to the reviewer comments have significantly improved our study. We thank you for your time and look forward to your response.

Asuka Nakajima, Yasushi Shimo, and the Co-authors

2nd Editorial Decision 21 August 2017

Dear Dr. Shimo,

Your revised manuscript was re-evaluated by external reviewers as well as by the Section Editor, Dr. Yoland Smith and ourselves. We are pleased to inform you that we expect that it will be acceptable for publication in EJN following further revision.

Although both authors were satisfied with the changes you've made to the original version of the manuscript, they both raised some additional minor concerns that must be taken care of before acceptance of the paper for publication. Please, make sure to carefully address each of their comments in your revised version. Particularly important, as pointed out by reviewer 2, is the quality of the figures. Please ensure that you are consistent with the use of fonts and ensure that the lettering is large enough to be easily read. Figure 10 needs replacing with a higher quality version; do you have the correct 'Y' axis? In the legend to this figure, SNT is used rather than STN and the abbreviation FSCV is not needed. Concerning the FSCV, it is not clear in the text nor figure how many times this experiment was performed. Please also correct the places where you have referred to P as equal to zero.

If you are able to respond fully to the points raised, we shall be pleased to receive a revision of your paper within 30 days.

Thank you for submitting your work to EJN.

Kind regards,

Paul Bolam & John Foxe
co-Editors in Chief, EJN

Reviews:

Reviewer: 1 (Paul Apicella, Aix Marseille Université, France)

Comments to the Author
I think that the present version of the paper is still fraught with some inaccuracies and inappropriate statements. It needs further improvements.

1. Intro p.7, « PANs are GABAergic neurons which project mainly to globus pallidus external »

2. Intro p.7, « TANs are cholinergic interneurons that send output to influence GABAergic projection neurons within the striatum »

3. Intro p.7, « … one of these studies (Kita et al., 2005) studied the effect of STN stimulation on TAN activity, investigating with a small number of TANs using different from clinical stimulation parameters. » The end of this sentence is not correct. The justification provided by the authors for choosing to study the striatal TAN population does not seem to me to be particularly convincing.

4. Intro p.8, « For these reasons, it is important to explore the effect of… »

5. Results. The new Figure 9 is quite enigmatic to me. Are they the putamen (monkey S) ? and caudate nucleus (monkey C) ? The authors should provide a clearer illustration showing both striatal

nuclei (if possible with the location of adjacent GP) with headings of the different structures. Please, add a comment in the Results (p.21) about the distribution of the recorded TANs. Are they in the motor and/or associative parts of the dorsal striatum ?

6. Figure 3, caption, « Suppression of tonic firing was greatest at 130 Hz » The heading indicates 120 Hz.

7. Methods p.9, The new sentence added sounds awkward « The head of the animal was fixed, but the body could move freely. » Maybe I did not make myself clear enough when I asked about description of the behavior during HFS : If the monkey « moves freely », then what it is doing during HFS ? Is it expecting any rewarding event to help to keep it quite ? Is there any constraint on its hand movements ?

8. While reading the authors' justification for not introducing PAN recordings to supplement TAN data (Reviewer 2, Point 2), I discovered that the neuronal recordings would have been collected under sedation ? « Technically, PANs are difficult to identify using our experimental conditions because PANs rarely fire in sedated monkeys. PANs fire phasically during behavior… » Can you just clarify that for me ?

9. Discussion p.23, « Further, recent anatomical studies showed that a direct projection from the STN to the striatum (Koshimizu et al., 2012) or ventral thalamus (Rico et al., 2010). » The sentence is not correct.

10. Some statements in the Discussion are rather inadequate or not sufficiently precise.
« The thalamus sends a massive glutamatergic axonal projection to both PANs and GABAergic interneurons in the striatum (Smith et al., 2014) » (p.24). What about the thalamic projection to striatal ChIs which is known to be particularly prominent ?
« The other is GABAergic interneurons (Tepper et al., 2010). GABAergic interneurons fire tonically at higher rates than do TANs, and send their axons to TANs ». Are the authors aware that striatal GABAergic interneurons fall into several distinct subtypes ? What exactly the authors were talking about ?

Reviewer: 2 (Natalie Doig, University of Oxford, UK)

Comments to the Author
I believe that the authors have addressed the reviewers' concerns since the first version of the manuscript.

This study addresses an important aspect of the relationship between DBS and the firing of TANs and I believe will be of interest to readers of this journal and the wider scientific community.

I have 2 minor points...
1) The use of the term 'cholinergic interneuron' should be used cautiously - 'putative cholinergic interneurons' or 'tonically active neurons' is more appropriate since the neurochemical composition of the neurons is never examined.
2) The figures are still not of as high quality as would be expected for publication in this journal, particularly the voltammetry figure which could be displayed in a much more reader-friendly format. However I leave this issue to the discretion of the editors.

Authors' Response 19 September 2017

Dear Editor
We thank the reviewers for their careful reading of our manuscript and their valuable comments to improve the quality of our manuscript. We have revised the manuscript based on the reviewers' comments. We added acknowledgements of the National BioResource Project (NBRP Japanese macaques), Japan, which

provided two monkeys for this project; and Dr Thomas Wichmann, who provided the MATLAB code that he and Y.S created to calculate the burst index.

Response to Editors
Please ensure that you are consistent with the use of fonts and ensure that the lettering is large enough to be easily read.
Response:
We enlarged the fonts in the figures.

Figure 10 needs replacing with a higher quality version; do you have the correct 'Y' axis? In the legend to this figure, SNT is used rather than STN and the abbreviation FSCV is not needed.
Response:
We replaced Figure 10 and corrected the Y-axis label. We also changed “SNT” to “STN” and deleted “fast scan cyclic voltammetry”.

Concerning the FSCV, it is not clear in the text nor figure how many times this experiment was performed.
Response:
We added the number of times the FSCV experiment was performed.

Please also correct the places where you have referred to P as equal to zero.
Response:
We changed “P=0.000” to “P<0.001”.

Response to the Reviewers
Reviewer 1
1. Intro p.7, « PANs are GABAergic neurons which project mainly to globus pallidus external »

2. Intro p.7, « TANs are cholinergic interneurons that send output to influence GABAergic projection neurons within the striatum »

3. Intro p.7, « … one of these studies (Kita et al., 2005) studied the effect of STN stimulation on TAN activity, investigating with a small number of TANs using different from clinical stimulation parameters. » The end of this sentence is not correct. The justification provided by the authors for choosing to study the striatal TAN population does not seem to me to be particularly convincing.

4. Intro p.8, « For these reasons, it is important to explore the effect of… »
Response:
We corrected all the sentences that Reviewer notes above. In response to comment 3, we added and changed some sentences to relay the importance of exploring TAN activity during STN or GPi HFS. The revised text is “Several studies have investigated the effects of STN stimulation on PAN activity using electrophysiology or microarrays (Kita *et al.*, 2005; Gubellini *et al.*, 2006; Visanji *et al.*, 2015), but only one study explored the effect of STN stimulation on TAN activity (Kita *et al.*, 2005); this study was performed in only a small population of neurons and used a 100-Hz stimulation frequency and an electric pulse duration of 300 µs (or a single electric pulse lasting 300 µs) as the stimulation parameters, which are completely different from the stimulation parameters used in clinical settings. Moreover, no study has investigated the relationships between GPi stimulation and TAN activity, and the mechanisms responsible for the effect of STN or GPi stimulation on TAN activity are unknown. ”

5. Results. The new Figure 9 is quite enigmatic to me. Are they the putamen (monkey S) ? and

caudate nucleus (monkey C) ? The authors should provide a clearer illustration showing both striatal nuclei (if possible with the location of adjacent GP) with headings of the different structures. Please, add a comment in the Results (p.21) about the distribution of the recorded TANs. Are they in the motor and/or associative parts of the dorsal striatum ?

Response:

We have made a new figure in which we plotted the recording sites on the atlas that we used to choose the chamber location before implanting and mapping the striatum for each monkey. Our approach is detailed in the Methods section. The region showing a drug response was the dorso-lateral part of the putamen, which has sensory-motor functions. We added the sentence "The responsive TANs were mainly located in the dorsolateral region of the putamen and in the region that is posterior to the anterior commissure; these areas correspond to known sensorimotor areas of the putamen (Parent & Hazrati, 1995; Haber, 2016; Marche *et al.*, 2017). "

6. Figure 3, caption, « Suppression of tonic firing was greatest at 130 Hz » The heading indicates 120 Hz.

Response:

We corrected this point.

7. Methods p.9, The new sentence added sounds awkward « The head of the animal was fixed, but the body could move freely. » Maybe I did not make myself clear enough when I asked about description of the behavior during HFS : If the monkey « moves freely », then what it is doing during HFS ? Is it expecting any rewarding event to help to keep it quite ? Is there any constraint on its hand movements ?

Response:

We changed the sentence to "The head of the animal was fixed in place using head holders, as detailed in the following section, but the rest of the body was not restrained." The bodies of the animals were not restrained in any way; we relied on the training and mild sedation to keep them still.

8. While reading the authors' justification for not introducing PAN recordings to supplement TAN data (Reviewer 2, Point 2), I discovered that the neuronal recordings would have been collected under sedation ? « Technically, PANs are difficult to identify using our experimental conditions because PANs rarely fire in sedated monkeys. PANs fire phasically during behavior… » Can you just clarify that for me ?

Response:

These details are included in the Methods section as: "During each recording session, monkeys were lightly sedated with ketamine hydrochloride (Daiichi Sankyo Propharma Co., Tokyo, Japan) (0.5 to 0.75 mg, i.m.) and medetomidine hydrochloride (Nippon Zenyaku Kogyo Co., Fukushima, Japan) (0.01 to 0.015 mg, i.m.)."

9. Discussion p.23, « Further, recent anatomical studies showed that a direct projection from the STN to the striatum (Koshimizu et al., 2012) or ventral thalamus (Rico et al., 2010). » The sentence is not correct.

Response:

We changed the sentence to "Further, recent anatomical studies identified a direct projection from the STN to the striatum (Koshimizu et al., 2012) and the ventral thalamus (Rico et al., 2010)."

10. Some statements in the Discussion are rather inadequate or not sufficiently precise. « The thalamus sends a massive glutamatergic axonal projection to both PANs and GABAergic interneurons in the striatum (Smith et al., 2014) » (p.24). What about the thalamic projection to striatal ChIs which is known to be particularly prominent ?

Response:

We thank the reviewer for these important suggestions.

EJN European Journal of Neuroscience FENS Federation of European Neuroscience Societies

We changed the sentence to "and it is well known that the thalamus sends a massive glutamatergic axonal projection to the striatum (Smith et al., 2014)." We also added a sentence about the role of glutamatergic input from the thalamus to TANs: "Further, TANs receive glutamatergic input from the centromedian-parafascicular complex of the thalamus. This input has an important role in evoking the characteristic response pattern of TANs: a pause followed by an excitatory rebound response (Matsumoto et al 2001). Further experiments are needed to explore the role of glutamatergic input on TAN activity in relation to STN- or GPi-HFS."

« The other is GABAergic interneurons (Tepper et al., 2010). GABAergic interneurons fire tonically at higher rates than do TANs, and send their axons to TANs ». Are the authors aware that striatal GABAergic interneurons fall into several distinct subtypes ? What exactly the authors were talking about ?

Response:

We changed the sentence to "The other is GABAergic interneurons, several types of which are present in the striatum (Tepper *et al.*, 2010)."

Reviewer 2

1) The use of the term 'cholinergic interneuron' should be used cautiously - 'putative cholinergic interneurons' or 'tonically active neurons' is more appropriate since the neurochemical composition of the neurons is never examined.

Response:

We made the appropriate changes. We also changed the title from "Subthalamic Nucleus and Globus Pallidus Interna Influence Firing of Striatal Cholinergic Neurons by Different Mechanisms." to "Subthalamic Nucleus and Globus Pallidus Interna Influence Firing of Tonically Active Neurons in the Primate Striatum through Different Mechanisms."

2) The figures are still not of as high quality as would be expected for publication in this journal, particularly the voltammetry figure which could be displayed in a much more reader-friendly format. However I leave this issue to the discretion of the editors.

Response:

We have replaced Figure 10.
